# Design of Acoustic Absorbing Structures for Mercurous Halide-Based Acousto-Optic Tunable Filters

**DOI:** 10.3390/ma17225606

**Published:** 2024-11-16

**Authors:** Shujing Sun, Huijie Zhao, Qi Guo, Yijie Wang

**Affiliations:** 1 School of Instrumentation and Opto-Electronic Engineering, Beihang University, Beijing 100191, China; sunshujing@buaa.edu.cn (S.S.); hjzhao@buaa.edu.cn (H.Z.); wangyjrd@buaa.edu.cn (Y.W.); 2 Qingdao Research Institute, Beihang University, Qingdao 266104, China; 3 Institute of Artificial Intelligence, Beihang University, Beijing 100191, China; 4 Aerospace Optical-Microwave Integrated Precision Intelligent Sensing Key Laboratory of Ministry of Industry and Information Technology, Beihang University, Beijing 100191, China

**Keywords:** mercurous halides, AOTF, diffraction efficiency, absorber

## Abstract

For the acousto-optic tunable filter (AOTF)-based spectral imaging systems, the diffraction efficiency of the AOTF is a primary factor affecting system throughput. Moreover, the distribution of the acoustic field within the AOTF fundamentally determines the device’s diffraction efficiency. Thus, the design of an AOTF device including a transducer and absorber to achieve a uniform acoustic field distribution plays an important role in improving diffraction efficiency. This study proposed an acoustic absorbing structure using mercurous halide crystals’ strong acoustic anisotropy to realize the conversion from shear horizontal wave to surface wave at the boundary and rapid dissipation. Snell’s law for acoustically anisotropic media is employed to design the angle of the acoustic absorbing structure. Experiments of mercurous halide-based AOTF demonstrate that this absorbing structure can effectively enhance diffraction efficiency.

## 1. Introduction

The essence of acousto-optic interaction is the photoelastic effect within the acousto-optic medium, where the elastic strain induced by the acoustic field causes the light to be deflected, diffracted, or have its energy altered [1]. Acousto-optic tunable filters (AOTFs) utilize the interaction between acoustic waves and incident light within an acousto-optic medium under the Bragg condition to achieve acousto-optic diffraction effects. Due to their advantages, such as rapid band switching, continuous wavelength tuning, flexible switching capabilities, and the absence of moving mechanical parts, AOTFs have found wide applications in recent years in fields like space exploration [2,3], spectral microscopy [4], 3D imaging [5], and laser control [6].

The acousto-optic medium used for AOTFs is diverse, primarily including crystals such as tellurium dioxide (TeO_2_), tellurium (Te), and mercury halides (Hg_2_Br_2_ and Hg_2_Cl_2_) [7,8,9]. Among these, tellurium dioxide (TeO_2_) is a mature commercial acousto-optic crystal. As AOTF spectral detection advances into the middle to long wavelength ranges, mercury halide crystals are considered important materials due to their high diffraction efficiency and wide transmission range [10,11,12]. In an AOTF-based spectral imaging system, diffraction efficiency and transmittance are critical factors influencing system performance [13,14]. Initial results have been achieved using mercurous bromide (Hg_2_Br_2_) crystals for long-wave AOTFs [14]. Due to the low driving frequencies of mercurous halide (Hg_2_X_2_) AOTFs in the mid-to-long wavelength range [14,15], the minimal acoustic attenuation from interface reflection significantly disrupts the acoustic field distribution within the AOTF [16]. However, the acoustic field distribution within the AOTF significantly determines its diffraction efficiency [17], making the design of the acoustic-absorbing structure crucial.

The design of the absorption structure for an acousto-optic tunable filter (AOTF) primarily encompasses reflective and transmissive absorption methods. Recent advancements in the study of reflective acoustic fields within AOTFs [18,19,20,21,22] have led to the widespread adoption of reflective absorption structures [1,23]. These structures, while simple in design, are confined to high-frequency driving conditions where significant acoustic loss occurs. Transmissive acoustic absorption in AOTFs is typically achieved by attaching soft metals like indium and lead to the absorbing surface [24,25]. However, the chemically reactive nature of mercurous halides (Hg_2_X_2_) can corrode these acoustic-absorbing materials [9,15], rendering them unsuitable for use in mercurous halide (Hg_2_X_2_) AOTFs. Consequently, a specialized acoustic-absorbing structure design is necessary for these types of AOTFs.

This paper analyzes the acoustic propagation characteristics at the interfaces of mercury halide crystals, which exhibit acoustic anisotropy. By applying Snell’s Law to calculate the direction of reflected acoustic phase velocity, the direction of reflected acoustic wave energy is determined. Through designing the angle of the interface, the acoustic body waves are converted into surface waves at the absorption body of the AOTF where mercury halide is used as the acousto-optic crystal. Through the design and fabrication of an acoustic absorption structure for mercurous bromide (Hg_2_Br_2_) AOTF, mercurous chloride (Hg_2_Cl_2_) crystals were selected as the acoustic absorption crystal material. By comparing the power response curves of the Hg_2_Br_2_ AOTF before and after installing the acoustic absorption structure, a significant improvement in the AOTF diffraction efficiency was observed. Furthermore, the power response curve of the AOTF with the installed acoustic absorption structure closely matched the simulation data under conditions of complete acoustic energy absorption.

## 2. Materials and Methods

**Figure 1:**
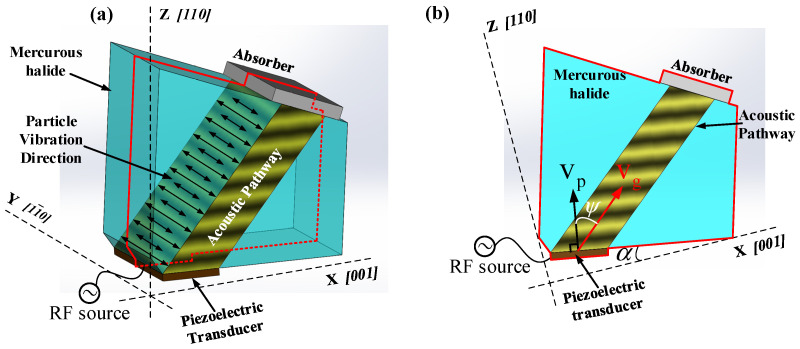
(**a**) AOTF three-dimensional structure diagram; (**b**) a sectional view of the dashed line position in the AOTF three-dimensional diagram.

The construction of the ultrasonic field in the AOTF device primarily consists of three components: the piezoelectric transducer, the acousto-optic medium, and the absorber, as illustrated in Figure 1a. The piezoelectric transducer generates vibrations driven by a radio frequency signal, producing horizontal shear waves with particle displacement perpendicular to the XZ plane. These ultrasonic waves transmit through the acousto-optic medium and are ultimately absorbed by the absorber. To describe the distribution of the ultrasonic field in acousto-optic medium, the crystallographic coordinates X, Y, and Z axes correspond to the crystal’s ***[001]*** axis, ***[***$1\bar{1}0$***]*** axis, and ***[110]*** axis, respectively. Figure 1b shows the YZ cross-section of the AOTF highlighted by the red frame in Figure 1a, where the angle between the transducer and the X-axis is referred to as the transducer cut angle α. Due to the strong acoustic anisotropy of the acousto-optic medium, there exists an acoustic walk-off angle ψ between the phase velocity and group velocity of the acoustic field within the medium. The purpose of the absorbing structure in the AOTF is to absorb the traveling acoustic wave within the acousto-optic medium, preventing reflected acoustic field energy from interfering with the bulk grating, thus ensuring a stable distribution of the acousto-optic grating within the medium.

In the acousto-optic medium, the propagation direction of the acoustic energy aligns with the group velocity. To determine the angular relationship between the phase velocity and group velocity, it is necessary to solve the Poynting theorem for the acoustic field with given material parameters of the medium. The speed of acoustic *v* in an acoustically anisotropic medium varies with direction, and can be calculated using the fundamental equations of ultrasonic bulk waves [1].

(1) $$(LcL^{\prime }-\rho v^{2}\delta_{ij})u_{j}=0$$

Here, L is the matrix representing the direction of the acoustic wave vector, $L^{\prime }$ is the transpose of L, and $\delta_{ij}$ is the Kronecker delta operator, which equals 1 when i = j and 0 otherwise. In this paper, $u_{j}$ represents the particle displacement due to the ultrasonic vibration, *ρ* is the density, and c is the matrix of the elastic stiffness coefficients. TeO_2_, Hg_2_Br_2_, and Hg_2_Cl_2_ all belong to the tetragonal crystal category, with the elastic stiffness coefficients represented by

(2) $$c=\left(\begin{array}{cccccc} c_{11} & c_{12} & c_{13} & 0 & 0 & 0\\ c_{12} & c_{11} & c_{13} & 0 & 0 & 0\\ c_{13} & c_{13} & c_{33} & 0 & 0 & 0\\ 0 & 0 & 0 & c_{44} & 0 & 0\\ 0 & 0 & 0 & 0 & c_{44} & 0\\ 0 & 0 & 0 & 0 & 0 & c_{66} \end{array}\right)$$

Based on the literature and measured data, the specific values of the elastic stiffness coefficients for TeO_2_, Hg_2_Br_2_, and Hg_2_Cl_2_ can be summarized as shown in Table 1. The elastic stiffness coefficients for TeO_2_ and Hg_2_Cl_2_ are provided by literature data [9], while the data for Hg_2_Br_2_ are obtained from experimental tests [26].

**Figure 2:**
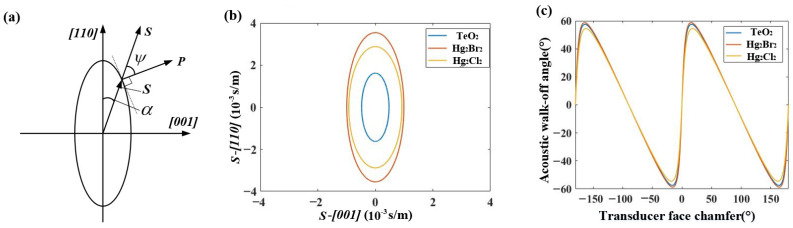
(**a**) Schematic diagram of the Poynting vector direction; (**b**) slowness curves in the XZ plane for TeO_2_, Hg_2_Br_2_, and Hg_2_Cl_2_; (**c**) acoustic walk-off angles for TeO_2_, Hg_2_Br_2_, and Hg_2_Cl_2_ at different angles.

The group velocity, which represents the direction of acoustic wave energy, is aligned with the Poynting vector ***P***. According to Figure 2a, the direction of the Poynting vector ***P*** is the normal to the tangent at the intersection of the slowness vector s and the slowness curve. The direction of the slowness vector s is the same as that of the phase velocity $v_{p}$, and its magnitude is the reciprocal of the phase velocity. At this point, the angle between the Poynting vector ***P*** and the slowness vector s is the acoustic walk-off angle ψ. Figure 2b shows the slowness curves for TeO_2_, Hg_2_Br_2_, and Hg_2_Cl_2_ crystals at the acousto-optic interaction surface, while Figure 2c illustrates the distribution of the acoustic walk-off angles for different transducer cut angles.

Based on the direction of acoustic propagation, the position can be determined to design the absorbing interface. The absorbing structure primarily considers two aspects: acoustic energy reflection and acoustic energy transmission. Acoustic energy reflection involves controlling the reflected acoustic energy to deviate from the position of the acousto-optic grating, which is mainly applicable to high-frequency acoustic fields that decay rapidly. Mercurous halide (Hg_2_X_2_), with its superior optical transmission in the mid-to-long-wave infrared range, corresponds to a low-frequency acoustic field, where reflected acoustic waves can undergo multiple reflections within the crystal, thereby interfering with the uniformity of the bulk grating.

The primary aspect of acoustic energy transmission refers to the transfer of acoustic energy at an interface, where the energy from the main acoustic field is conveyed into another medium, such as acoustic-absorbing materials like indium or lead [24,25]. Due to the reactive chemical properties of Hg_2_X_2_, it can easily interact with the bonding medium. Therefore, while searching for suitable absorbing materials, research can also focus on the critical angle conditions for acoustic energy propagation at the boundary.

**Figure 3:**
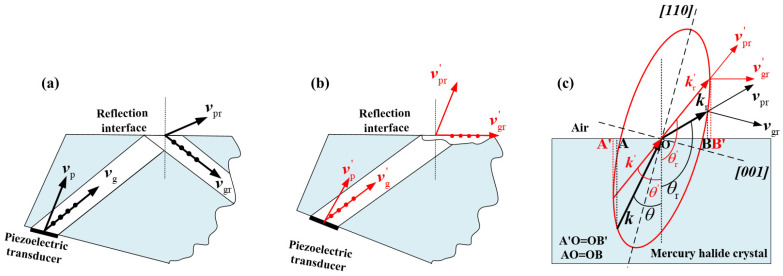
(**a**) The schematic diagram of the reflected wave energy returning to the crystal interior; (**b**) the schematic diagram of the reflected wave energy propagating along the boundary; (**c**) the diagram illustrating the principle of Snell’s law at the interface.

Since the AOTF primarily uses SH waves in the XZ plane, there is no mode conversion during acoustic propagation across the boundary, meaning the reflected acoustic field remains as SH waves [19]. The phase velocities at the interface between two acoustically anisotropic mediums adhere to Snell’s law [18]. Unlike isotropic media, at the interface of anisotropic media, the phase velocity reflection angle can exceed 90°, meaning the phase velocity direction tilts toward the other medium, while the group velocity direction remains within 90°. Therefore, the acoustic energy continues to propagate within the acousto-optic crystal, as shown in Figure 3a.

Assuming the medium in contact with the acousto-optic crystal is air, the acoustic field energy is theoretically completely reflected back into the acousto-optic crystal. However, when the direction of the phase velocity reaches a critical angle relative to the boundary, a special condition occurs, as illustrated in Figure 3b. In this case, the calculated group velocity direction based on the phase velocity of the reflected acoustic field exceeds 90°, and since SH waves cannot propagate in air, the acoustic energy transmits along the boundary, forming surface acoustic waves.

Figure 3c illustrates the two scenarios of acoustic reflection shown in Figure 3a and Figure 3b through the acoustic wave vector curves. The wave vector curves can be derived from the slowness curve, where the relationship between slowness s and wave vector k is given by

(3) $$k=2\pi fs=\frac{2\pi f}{v_{p}}$$

Here, f is the driving frequency of the acoustic field. In Figure 3c, the medium at the acoustic reflection interface of the acousto-optic crystal is air. When the wave vector k is incident at an angle θ, the corresponding angle of the reflected wave vector is $\theta_{r}$, resulting in the reflected acoustic energy propagating within the acousto-optic crystal. Conversely, when the wave vector $k_{0}$ is incident at an angle $\theta_{0}$, the corresponding angle of the reflected wave vector is $\theta_{0r}$, allowing the reflected acoustic energy to propagate along the interface in the form of surface waves. Additionally, AO = OB and A_0_O = B_0_O conform to Snell’s law for acoustic propagation at the interface of anisotropic media.

(4) $$\frac{\left|\sin\theta \right|}{\left|k\right|}=\frac{\left|\sin\theta_{r}\right|}{\left|k_{r}\right|}=\frac{\left|\sin\theta_{t}\right|}{\left|k_{t}\right|}$$

Here, k, $k_{r}$, and $k_{t}$ represent the incident vector, reflected vector, and transmitted vector, respectively, while θ, $\theta_{r}$, and $\theta_{t}$ denote the wave vector incident angle, wave vector reflected angle, and wave vector transmitted angle, respectively.

**Figure 4:**
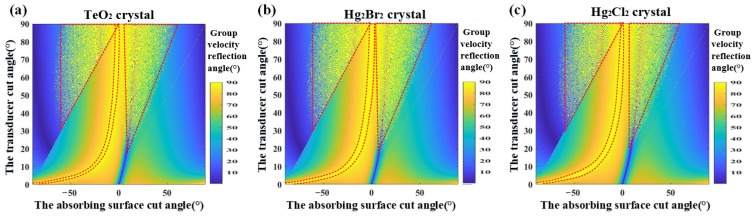
(**a**) The distribution diagram of the group velocity reflection angle of TeO_2_; (**b**) the distribution diagram of the group velocity reflection angle of Hg_2_Br_2_; (**c**) the distribution diagram of the group velocity reflection angle of Hg_2_Cl_2_.

Based on the schematic in Figure 3, the direction of the reflected phase velocity at the boundary is primarily determined by the acoustic incident angle and the cut angle of the absorbing surface. Based on the slowness distribution and acoustic walk-off angle data for the three crystals shown in Figure 2, we calculated the variation of the group velocity angle of the reflected acoustic field at the boundary as a function of the acoustic incidence angle and the angle of the absorbing surface, as shown in Figure 4. Here, the angle of the absorbing surface is defined as the angle between the absorbing boundary and the X-axis. Figure 4a–c correspond to TeO_2_, Hg_2_Br_2_, and Hg_2_Cl_2_, respectively.

When the acoustic incident angle ranges from 0° to 90°, and the absorbing surface cut angle ranges from −90° to 90° with clockwise as positive and counterclockwise as negative, the overall distribution of group velocity angles at the boundaries for TeO_2_, Hg_2_Br_2_, and Hg_2_Cl_2_ is similar. When the group velocity reflection angle reaches its maximum value of 90°, the reflected acoustic field propagates as surface acoustic waves and decays rapidly. Therefore, in Figure 4, the regions corresponding to a group velocity reflection angle of 90° for the three crystals are marked with red dashed lines. The triangular area requires a larger incident angle θ, while the strip-shaped region should be analyzed by considering both the acoustic incident angle and the absorbing surface cut angle.

Based on the above data, to achieve the conversion of ultrasonic bulk waves into surface waves, a smaller acoustic incident angle needs to be close to a 90° absorbing surface cut angle, which can lead to significant waste of the crystal. Therefore, it is necessary to increase the acoustic incident angle to reduce the absorbing surface cut angle. However, to ensure efficient acousto-optic diffraction, the transducer cut angle should not be too large, as this would lower the diffraction efficiency.

**Figure 5:**
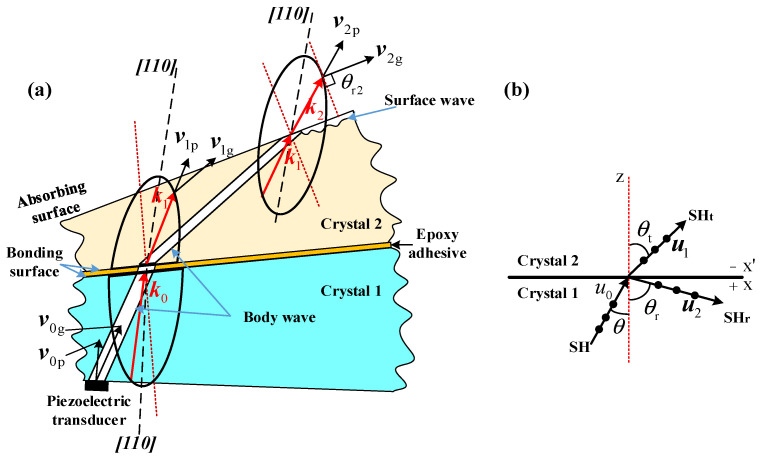
(**a**) Acoustic transmission and absorption diagram; (**b**) diagram of acoustic wave propagation through two crystals.

By combining the ideas of acoustic energy transmission and reflection, as shown in Figure 5, acoustic energy can be transmitted from the acousto-optic crystal through crystals of the same or similar materials. The other material does not undergo acousto-optic interaction, so there is no angle limitation, and it can be designed according to the data in Figure 4 to achieve the transformation of body waves to surface waves, thereby improving the acousto-optic diffraction performance of the AOTF and reducing the waste caused by the angle cutting of acousto-optic crystals.

In Figure 5a, crystal 2 is bonded to crystal 1 using epoxy resin (Huntsman, Genève, Switzerland). By designing and cutting the bonding surface and the absorbing surface angle of crystal 2, the group velocity reflection angle of the transmitted wave at the absorbing boundary of crystal 2 is set to 90°. The thickness of the epoxy layer should be much smaller than the acoustic wavelength. For Hg_2_X_2_, within the frequency range of 10–20 MHz, the adhesive thickness should be kept within 1 μm to avoid affecting the transmission of acoustic energy. The cured epoxy resin creates a rigid connection between the two crystals, ensuring that displacement and stress continuity are maintained at the interface during acoustic transmission [27].

(5) $$\left\{\begin{array}{l} u_{y+}=u_{y^{\prime }-}\\ \tau_{xy+}=\tau_{x^{\prime }y^{\prime }-} \end{array}\right.$$

Here, $u_{y+}$ and $u_{y^{\prime }-}$ represent the total displacements at the bonding surfaces of crystal 1 and crystal 2, respectively, while $\tau_{xy+}$ and $\tau_{x^{\prime }y^{\prime }-}$ represent the stresses at the bonding surfaces of crystal 1 and crystal 2, respectively. This ensures the continuity of displacement and stress under the condition of a rigid connection. Based on Equation (5), the displacements of the SH wave, SH_t_ wave, and SH_r_ wave, as shown in Figure 5b, are represented as $u_{0}$, $u_{1}$ and $u_{2}$, respectively. The transmission coefficient $\Gamma_{t}$ at the interface can be obtained from the amplitude ratio of $u_{1}$ and $u_{0}$.

(6) $$\left\{\begin{array}{l} u_{0}=u_{y+}-u_{2}=Ae^{-ik_{0}\cdot r0}\\ u_{1}=u_{y-}=Be^{-ik_{1}\cdot r1}\\ \Gamma_{t}=\frac{B}{A} \end{array}\right.$$

Here, $k_{0}$ and $k_{1}$ represent the wave vectors of the incident and transmitted acoustic waves, respectively, while r0 and r1 denote the acoustic propagation distances in the XY plane for the incident and transmitted waves.

From this, the transfer function for SH waves at the interface can be derived, allowing for the calculation of the transmission coefficient under different incident conditions. Notably, when the displacement amplitudes of SH waves and SH**_t_** waves are equal, the transmission coefficient is 1, indicating that there is no reflected wave energy.

## 3. Results and Discussion

In order to verify the effectiveness of the absorption structure based on Hg_2_X_2_ crystals in AOTF, an AOTF using Hg_2_Br_2_ as the acousto-optic crystal was prepared. Lithium niobate was cut in the X orientation to generate shear waves polarized along the Y-axis in Hg_2_Br_2_. The transducer has a thickness of 200 μm, and after transducer impedance matching, the driving frequency range is 8–12 MHz. Based on actual device testing with incident light at a wavelength of 632 nm, the center frequency is 9.5 MHz. The transducer cut angle for Hg_2_Br_2_ is 0°, while the absorbing surface cut angle is −10°. The dimensions of the Hg_2_Br_2_ crystal are 17 mm along the X-axis and 10 mm along the Y-axis, while the transducer measures 8 mm along both the X-axis and Y-axis.

**Figure 6:**
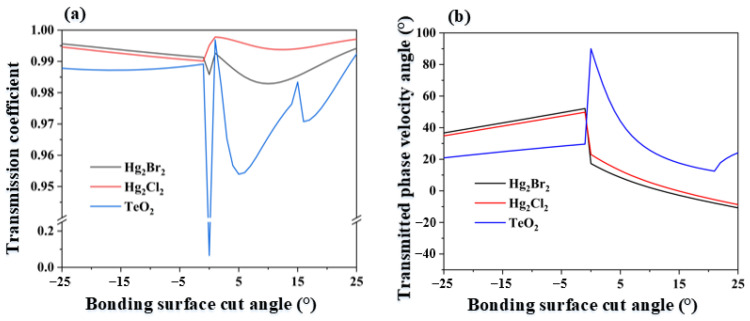
(**a**) Transmission coefficient distribution of Hg_2_Br_2_ with Hg_2_Br_2_, Hg_2_Cl_2_, and TeO_2_; (**b**) phase velocity/transmission angle distribution curves for Hg_2_Br_2_, Hg_2_Cl_2_, and TeO_2_.

To facilitate the conversion of ultrasonic bulk waves to surface waves, the 1 μm thick epoxy resin layer was used to bond Hg_2_Br_2_ with other media, allowing acoustic energy in the Hg_2_Br_2_ to be transmitted to the other media. Common materials such as TeO_2_ and Hg_2_Cl_2_ have similar parameters and acoustic impedance to Hg_2_Br_2_ and do not chemically react with it. Therefore, the transmission coefficients and phase velocity transmission angles at the interface between Hg_2_Br_2_ and the three kinds of crystals (TeO_2_, Hg_2_Br_2_, and Hg_2_Cl_2_) were calculated, as shown in Figure 6. Figure 6a presents the transmission coefficient variation curve for the three crystals with a bonding surface cut angle ranging from −25° to 25°, while Figure 6b shows the angle between the phase velocity of the transmitted acoustic wave and the Z-axis within the crystal.

From Figure 6a, it can be seen that Hg_2_Cl_2_ exhibits relatively stable acoustic transmission coefficients within the bonding angle range, with an overall transmission coefficient exceeding 0.99. Therefore, Hg_2_Cl_2_ is selected as the absorbing material. Based on Figure 6b, a bonding surface cut angle of 3° is chosen, resulting in a phase velocity angle of 16.5° within the absorbing crystal. According to the group velocity reflection angle distribution for Hg_2_Cl_2_ shown in Figure 4c, the absorbing surface cut angle can be set to 17°, thereby determining the material and angles for the absorbing crystal.

**Figure 7:**
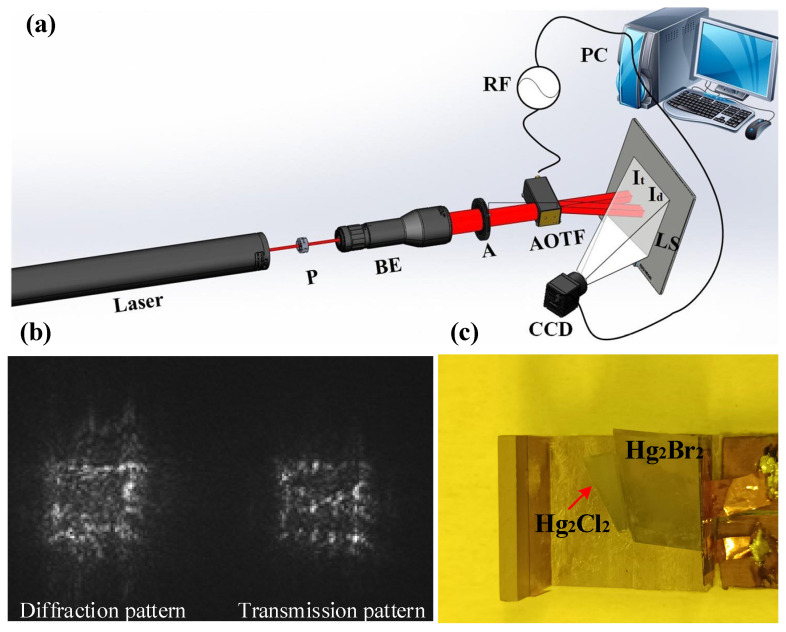
(**a**) Acoustic-optical diffraction test optical path diagram. P is the polarizer, BE is the beam expander, A is the aperture, RF is the radio frequency signal, I_t_ is the transmitted image, I_d_ is the diffracted image, LS is the light screen, and CCD is the charge-coupled device; (**b**) measured images of transmitted and diffracted images; (**c**) the photograph of the tested Hg_2_Br_2_ AOTF.

To verify the effectiveness of the absorbing structure, acoustic-optic diffraction tests were first conducted on the Hg_2_Br_2_ AOTF without the Hg_2_Cl_2_ absorber. The optical setup for the test is shown in Figure 7a, where a 632.8 nm wavelength He-Ne laser (Daheng Optics, Beijing, China) passes through a polarizer P (Thorlabs, Newton, NJ, USA) to produce ordinary polarized light [28]. After expanding the beam with a beam expander BE (Thorlabs, Newton, NJ, USA), it passes through an aperture A (Daheng Optics, Beijing, China) to achieve parallel light incidence on the AOTF’s optical surface at an incident angle of 12° and a driving frequency of 9.5 MHz. Acoustic-optic diffraction through the AOTF results in transmitted and diffracted images on a light screen, which were captured using a CCD (Basler, Arensburg, Germany) under different driving power levels. The transmitted and diffracted images are illustrated in Figure 7b. After bonding the Hg_2_Cl_2_ absorbing body to the Hg_2_Br_2_ AOTF, and once the epoxy resin has fully cured, the above tests were repeated. The main portion of the Hg_2_Br_2_ AOTF after bonding with the absorbing body is shown in Figure 7c.

**Figure 8:**
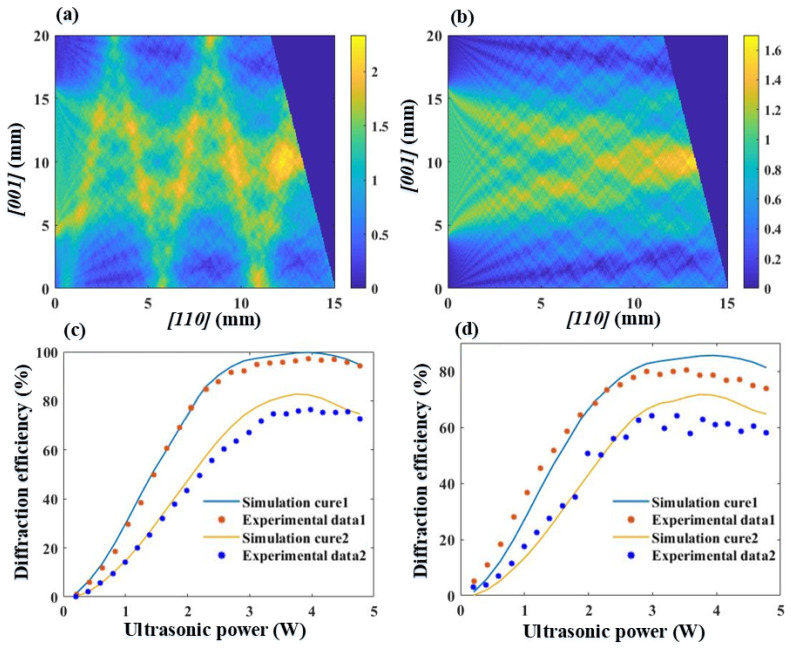
(**a**) The simulation diagram of the amplitude distribution of the acoustic field in mercuric bromide without absorber materials; (**b**) the simulation diagram of the amplitude distribution of the acoustic field in mercuric bromide with absorber materials; (**c**) the variation curves of single-point diffraction efficiency before and after the application of adhesive absorber materials with increasing power. Simulation Curves 1 and 2 represent simulation results, while Experimental Data 1 and 2 represent measured results; (**d**) the variation curves of image diffraction efficiency before and after the application of adhesive absorber materials with increasing power. Simulation Curves 1 and 2 represent simulation results, while Experimental Data 1 and 2 represent measured results.

Through simulation analysis of the acoustic field distribution within the Hg_2_Br_2_ crystal before and after the addition of the absorbing body [29,30,31], the amplitude distributions of the acoustic field in Hg_2_Br_2_ are obtained, as shown in Figure 8a,b, where the transducer is positioned at the left boundary. Using the numerical calculation method for acousto-optic interaction within an AOTF [28], the acoustic field distribution data within the acousto-optic crystal of an AOTF, both with and without an absorbing structure, are applied to the acousto-optic coupling equations. This enables the numerical calculation of the amplitude variations of the transmitted and diffracted light during the propagation of the incident light.

(7) $$\left\{\begin{array}{l} \frac{dC_{0}}{dx^{\prime }}=\frac{q_{0}(x^{\prime },y^{\prime },z^{\prime })}{2\cos\theta_{0}}C_{1}\exp\left\{i[\frac{\Delta kx^{\prime }}{\cos\theta_{1}}-\Phi (x^{\prime },y^{\prime },z^{\prime })]\right\}\\ \frac{dC_{1}}{dx^{\prime }}=-\frac{q_{1}(x^{\prime },y^{\prime },z^{\prime })}{2\cos\theta_{1}}C_{0}\exp\left\{-i[\frac{\Delta kx^{\prime }}{\cos\theta_{0}}-\Phi (x^{\prime },y^{\prime },z^{\prime })]\right\} \end{array}\right.$$

Here, *C*_0_ and *C*_1_ are the relative amplitudes of the transmitted light and the diffracted light, respectively. ***Φ*** represents the acoustic field phase distribution within the AOTF for incident light. *θ*_0_ and $\theta_{1}$ represent the angles of the incident light and diffracted light with respect to the ***[001]*** axis, respectively. q is the AO coupling coefficient, and its value is proportional to the amplitude of the acoustic field. Δk represents the acousto-optic coupling momentum mismatch caused by the non-uniform distribution of the acoustic field. Thus, by adjusting the driving power of the acoustic field, it is possible to calculate the simulated curves before and after adding the absorber to illustrate the trend in diffraction efficiency at the optical surface center and across the entire optical surface as the driving power increases.

In Figure 8c, Simulation Curve 1 and Simulation Curve 2 represent the simulation data for the diffraction efficiency at the center of the optical surface with and without the absorbing body, respectively, as driving power increases. Experimental Data 1 and Experimental Data 2 show the corresponding experimental data for the cases with and without the absorbing body. In Figure 8d, Simulation Curve 1 and Simulation Curve 2 display the simulation data for the overall diffraction efficiency of the optical surface with and without the absorbing body, respectively, while Experimental Data 1 and Experimental Data 2 present the experimental data for these two cases.

By comparing Figure 8a,b, it is evident that without the absorbing body, low-frequency acoustic energy undergoes multiple reflections within the acousto-optic medium and interferes with part of the energy in the acousto-optic grating. Consequently, Figure 8c,d clearly show that the diffraction efficiency without the absorbing body is significantly lower than that of the AOTF with the absorbing body.

The experimental data in Figure 8c indicate that after adding the absorbing structure, the maximum diffraction efficiency of the AOTF increased from the original 76.53% to 97.35%. Additionally, the simulation data align well with the experimental data, suggesting that the reflective acoustic field within the Hg_2_Br_2_ is essentially completely absorbed. In terms of the upward trend in diffraction efficiency, after adding the absorbing body, the diffraction efficiency tends to stabilize at a driving power of 2.3 W, whereas without the absorbing body, it requires nearly 3.1 W for the diffraction efficiency to stabilize.

The optical surface was not given a fine polish, resulting in less clear diffraction imaging and consequently affecting the overall diffraction efficiency. As shown in Figure 8d, the overall imaging diffraction efficiency is slightly lower than the single-point diffraction efficiency presented in Figure 8c. Additionally, the acousto-optic coupling simulation does not account for the acoustic energy loss in the transducer and the acousto-optic crystal under different driving power conditions. Consequently, the simulated diffraction efficiency is generally higher than the corresponding measured data.

## 4. Conclusions

This paper addresses the issue of the lack of an absorbing structure design for the mercurous halide (Hg_2_X_2_) AOTF. The application of Snell’s law to analyze the reflection patterns of acoustic fields at the commonly used acousto-optic crystals’ interfaces highlights the differences between the incident and reflection angles of phase velocity. Furthermore, we establish a critical reflection angle at the acoustic reflection boundary to allow reflected wave energy to propagate along the boundary, thus converting ultrasonic bulk waves into surface waves for effective acoustic absorption.

In order to make the acoustic wave energy reach the critical reflection angle, we bond angle-designed Hg_2_X_2_ crystals of the same or similar material as the acousto-optic crystal absorption surface to the AOTF. The experimental results show that introducing this absorption structure has increased the diffraction efficiency of the Hg_2_Br_2_ crystals-based AOTF from 76.53% to 97.35%. Additionally, the power required to achieve stable diffraction efficiency in the AOTF has been reduced by 0.8 W compared to the original setup, and the measured data results agree well with the simulation data. These results indicate that the proposed absorbing structure design can effectively improve the performance of Hg_2_X_2_ AOTF and reduce the waste of Hg_2_X_2_ crystals. This absorption structure design is expected to advance the application of Hg_2_X_2_ AOTF in mid-to-long wave detection systems.

This design of this acoustic absorption structure can aid in the research of high diffraction efficiency for Hg_2_X_2_ AOTFs. However, since the current study did not take into account the impact of acoustic field divergence on the design of the absorption structure, further detailed research in this area is necessary in the future.

## Figures and Tables

**Table 1:** The elastic stiffness constants of tellurium dioxide and mercurous halide crystals [9,26].

Crystal	Density ρ/(kg·m^−3^)	Elastic Stiffness Coefficient c/(10^10^ N·m^−2^)					
		$c_{11}$	$c_{12}$	$c_{13}$	$c_{33}$	$c_{44}$	$c_{66}$
TeO_2_	5990	5.57	5.12	2.18	10.58	2.65	6.59
Hg_2_Br_2_	7310	1.62	1.50	1.89	8.89	0.75	1.12
Hg_2_Cl_2_	7190	1.89	1.72	1.56	8.04	0.85	1.23

## Data Availability

Data are contained within the article.
